# Are Static and Dynamic Postural Balance Assessments Two Sides of the Same Coin? A Cross-Sectional Study in the Older Adults

**DOI:** 10.3389/fphys.2021.681370

**Published:** 2021-06-29

**Authors:** Alex Rizzato, Antonio Paoli, Marta Andretta, Francesca Vidorin, Giuseppe Marcolin

**Affiliations:** ^1^Department of Biomedical Sciences, University of Padova, Padova, Italy; ^2^School of Human Movement Sciences, University of Padova, Padova, Italy

**Keywords:** balance, postural control, geriatric assessment, dual-task, older adults

## Abstract

The aim of this study was to investigate if the combination of static and dynamic postural balance assessments gives more accurate indications on balance performance among healthy older adults. We also aimed at studying the effect of a dual-task condition on static and dynamic postural balance control. Fifty-seven healthy older adults (age = 73.2 ± 5.0 year, height = 1.66 ± 0.08 m, and body mass = 72.8 ± 13.8 kg) completed the study. Static and dynamic balance were assessed both in single-task and dual-task conditions through a force plate and an oscillating platform. The dominant handgrip strength was also measured with a dynamometer. Pearson’s correlation revealed non-statistically significant correlations between static and dynamic balance performance. The dual-task worsened the balance performance more in the dynamic (+147.8%) than in the static (+25.10%, +43.45%, and +72.93% for ellipse area, sway path, and AP oscillations, respectively) condition (*p* < 0.001). A weak correlation was found between dynamic balance performance and handgrip strength both in the single (*p* < 0.05; *r* = −0.264) and dual (*p* < 0.05; *r* = −0.302) task condition. The absence of correlations between static and dynamic balance performance suggests including both static and dynamic balance tests in the assessment of postural balance alterations among older adults. Since cognitive-interference tasks exacerbated the degradation of the postural control performance, dual-task condition should also be considered in the postural balance assessment.

## Introduction

Postural balance control has been defined as the ability of a subject to maintain the center of pressure (CoP) within the base of support to prevent falling (Winter et al., 1996). Traditionally, literature differentiates between static and dynamic balance conditions. The static condition is referred to balance under unperturbed environments such as quiet standing (Macpherson and Horak, 2013), while the dynamic condition is connected to the ability of the subject to react efficiently to the base of support displacements or to external mechanical stimuli (Paillard, 2019). The CoP displacement, derived from force platforms, is considered the most reliable output for postural balance control assessment under static conditions. Nevertheless, both static and dynamic postural control are crucial for the activities of daily living and are implicated in multiple scenarios of everyday life. Hence, the evaluation of the dynamic postural balance control is necessary besides the static one. Since the interaction of the postural control systems is complex, the assessment of postural balance control in a concise and holistic approach is demanding as well (Petró et al., 2017). On this point, Ringhof and Stein (2018) extended the traditional perspective considering balance as a general ability and reinforced the idea that dynamic balance tests are necessary and not interchangeable.

Consequently, the efficiency of the systems involved in postural balance control (i.e., visual, vestibular, and proprioceptive systems) is crucial for people of different ages. Since the mid-seventies, an increased postural sway in the older adults has been recognized (Hasselkus and Shambes, 1975; Baloh et al., 1998; Laughton et al., 2003), in association with a higher risk of falling (Fernie et al., 1982; Maki et al., 1994). Indeed, more than one-third of persons over 65 falls each year, and in half of such cases, falls are recurrent (Tinetti and Kumar, 2010). Hageman et al. (1995) found a larger area of sway in healthy older adults than in a younger group for all the studied conditions: eyes open, eyes closed, and with visual feedback. Similarly, Fernie et al. (1982) demonstrated a greater sway path velocity in older adults who had fallen once or several times in a year with respect to non-fallers. However, a review by Rubenstein (2006) reported that the fall-risk is more tightly associated with dynamic than static conditions. Moreover, Blake et al. (1988) identified tripping as the most recurrent fall-related event after a community survey on 1,042 individuals aged 65 and over. This age-dependent decrease in postural balance control has been interpreted as deterioration of sensory, motor, or cognitive systems (Woollacott and Shumway-Cook, 2002; Prado et al., 2007). Moreover, the reduced rate of force development in older adults has been associated with a lower capacity for neuromuscular response to control body balance (Izquierdo et al., 1999). Older fallers demonstrated a reduced contractile rate of force development than non-fallers (Fleming et al., 1991). Similarly, Paillard (2017b) hypothesized a relationship between lower extremity strength and postural performance (i.e., the stronger the muscles the better the postural performance), confirming the role of strength on postural balance control. A final aspect to consider is that many falls in older adults occurred when a secondary cognitive or motor task (i.e., dual-tasking) was performed (Tideiksaar, 1996; Hollman et al., 2007). Thus, the use of dual-task (DT) paradigms to predict falls among older adults is encouraged for its superiority over the employment of single-tasks (STs; Muir-Hunter and Wittwer, 2016; Bayot et al., 2020). The secondary task can be manual, discrimination and decision-making, mental tracking, verbal fluency, and working memory. Indeed, during dual-tasking, the two tasks reciprocally interfere with the performer’s attention overloading the cognitive sources (Ghai et al., 2017). Therefore, the introduction of a secondary cognitive task over the motor one (i.e., postural balance control) may help to understand the cognitive contribution involved in postural regulation (Pashler, 1994) both in static and dynamic conditions.

On the relevance of these previous investigations and considering that dynamic postural control has been recognized as important as the static postural control (Petró et al., 2017), the first aim of the present cross-sectional study was to investigate whether there was a relationship between static and dynamic postural balance performance in a group of healthy older adults. Moreover, considering the greater exposure to fall-risk of the older adults under dual-tasking condition, our second aim was to study if the addition of a cognitive-demanding task could equally affect static and dynamic postural balance control.

## Materials and Methods

### Subjects

Sixty-one healthy older adults were recruited from a Medical Center (CEMES, Data Medica group, Synlab S.p.A., Padova, Italy) after a clinical physiatric screening. Subjects over 65 years of age were eligible considering the following exclusion criteria: (i) lower-limb orthopedic injuries or falls in the last year; (ii) neurological pathologies; (iii) lower-limb joint replacement; (iv) low back pain; (v) the use of medications causing vertigo; (vi) sight, hearing, or vestibular disorders; and (vii) a score over 4 in the fall-risk questionnaire (FRQ), a self-assessment tool composed of 12 yes/no items, designed to screen older adults who are at risk of falling (Rubenstein, 2006; Rubenstein et al., 2011). The FRQ total score is obtained by summing the relative scores of the “yes” answers (two points to questions 1 and 2 and one point to the other 10 questions). A total score higher than 4 indicates a possible risk of fall. Out of the 61 subjects, three refused to perform trials in the DT condition and one did not perform the dynamic balance test because worried about the dynamic computerized platform. Therefore, 57 subjects (*F* = 30, *M* = 27; age = 73.2 ± 5.0 years, height = 1.66 ± 0.08 m, and mass = 72.8 ± 13.8 kg; FRQ score = 1.37 ± 1.59) completed the trials and were included in the data analysis.

### Experimental Design

The experimental protocol received approval from the Human Ethical Committee of the Department of Biomedical Sciences of the University of Padova (n° HEC-DSB/08–18) and adhered to the Declaration of Helsinki. All the subjects were informed about the methods and aims of the study, gave their written consent, and were free to renounce the study at any time. The week before testing, researchers organized a familiarization session explaining the scheduled program in detail to guarantee the correct execution.

We outlined a cross-sectional design in which two balance conditions, bipedal static (BS) and bipedal dynamic (BD) were tested both in ST and DT modality. During the ST modality, only the motor task had to be performed (i.e., static or dynamic standing); while during the DT modality, subjects were asked to perform an interfering cognitive task concurrently (i.e., counting aloud backward with a subtraction of 7 from a predetermined number). This DT modality requires both working memory and attention (Montero-Odasso et al., 2009). There was no instruction to prioritize either the motor or cognitive task. Static postural balance of each subject was assessed on a dynamometric platform (RGMD S.p.a., Genova, Italy; sample frequency: 100 Hz; CoP accuracy: 0.01 mm) and consisted of holding the same static upright position for three trials of 30 s each, according to the recommendations of Scoppa et al. (2013). Specifically, barefooted subjects were instructed to stand with extended legs, to place the arms along their sides naturally, and to gaze at a thin red line vertically placed on a white wall in front of them at a distance of 80 cm. The feet position on the force platform was standardized using a V-shaped layout, keeping a 7-cm distance between the heels and a wide open position of the tips of 30° between them according to the international society of posturography recommendations (Kapteyn et al., 1983).

**Figure 1:**
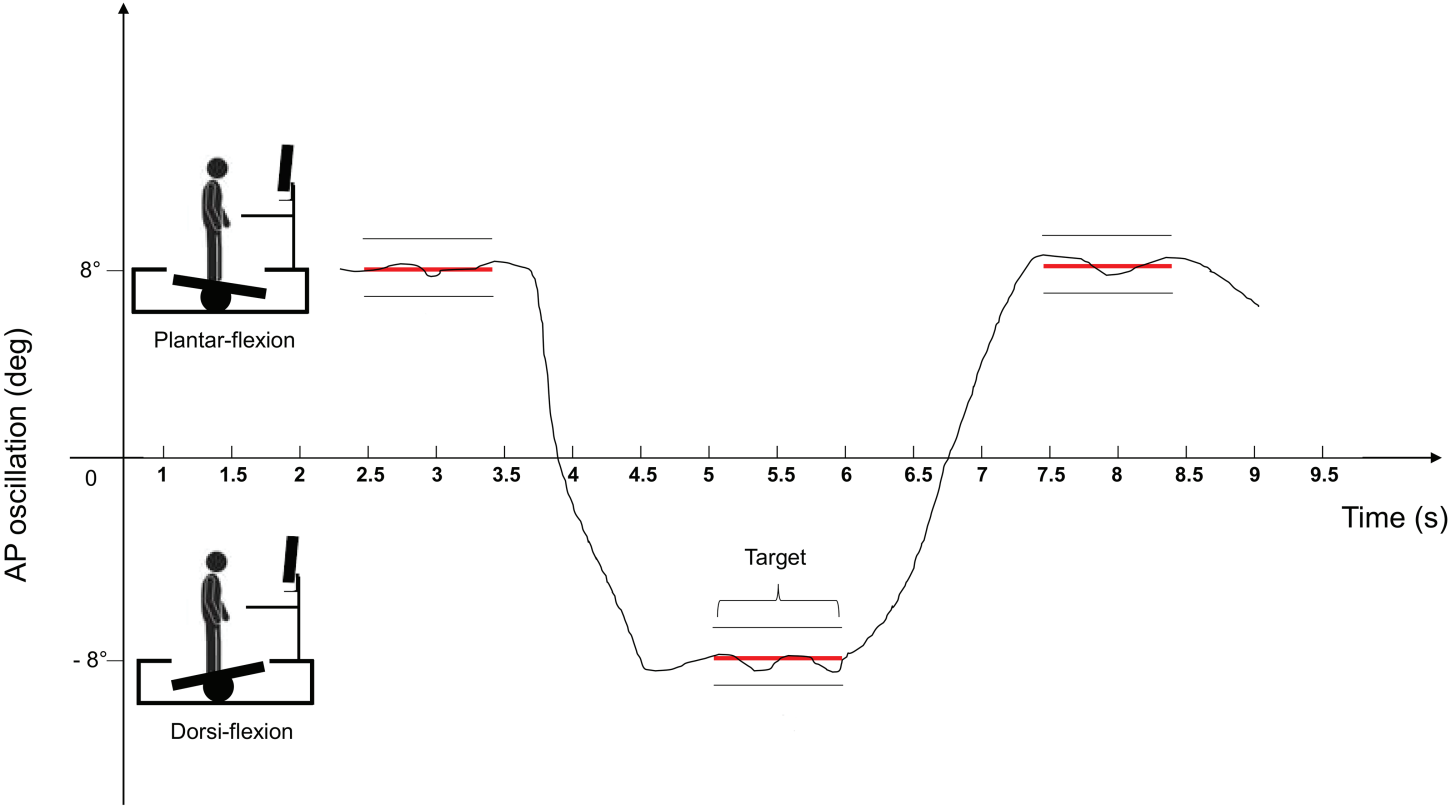
Graphical representation of the feedback given to the subjects during the dynamic postural balance test. The line on the screen represents the track of the angular signal that the subjects had to drive into superior and inferior targets moving the platform anterior-posteriorly through dorsi-flexion and plantar-flexion of the ankles.

Dynamic postural balance control was assessed on a medically approved (ISO 13485) computerized platform (mod. Prokin 252, Tecnobody, Bergamo, Italy; sample frequency: 20 Hz; angle accuracy: 0.5°) and consisting of three strain gauges set in a triangular position under a 55-cm diameter surface. The platform was calibrated according to the manufacturer’s guidelines. Subjects performed a specific dynamic balance task, having real-time feedback of the instant oscillation of the platform on a screen in front of them. Feet position was standardized as for the static test. They were required to drive the angle signal into predetermined superior or inferior gates, moving the platform anterior-posteriorly with dorsi-flexors and plantar-flexors muscles (Figure 1). The gates were set in correspondence to 8° of anterior and posterior inclination of the plate with respect to its transverse axis. A stay time of 1 s was set for each gate and the time interval between two consecutive gates was set to 1.5 s. The platform was blocked in the medio-lateral direction to allow only the anterior and posterior oscillations. In case of loss of balance, subjects could grab two handrails positioned in front of them. If it was the case, the trial was invalid. The task was demonstrated by one of the experimenters. Subsequently, subjects underwent a 5-min familiarization session. After a 3-min recovery, three trials lasting 30 s each were performed with opened eyes in the BS-ST, BS-DT, BD-ST, and BD-DT conditions. Dynamic tests were performed after the static ones. The DT and ST conditions were randomized both in the dynamic and static trials. The grip strength of the dominant hand was measured using a handgrip dynamometer (Saehan Corp®, SH5001, South Korea). Indeed, the strength of a person’s handgrip has been recognized as a valid measure of the overall muscle function and strength from age 50 onward (Steiber, 2016). Three consecutive measurements were made with subjects seated upright and with the elbow flexed at 90° (Bohannon et al., 2006).

### Data Analysis

In the static conditions, we considered the following classical stabilometric parameters calculated from the CoP trajectory: ellipse area (mm^2^), sway path (mm/s), and maximal AP oscillations (mm). The analysis was performed for each subject, extracting data from each trial concerning both BS-ST and BS-DT conditions. In the dynamic conditions, the computerized platform calculated an overall total stability index (TSI) as follow:

$$Totalstabilityindex\;\left(TSI\right)=\sqrt{\frac{\sum_{i=1}^{n}{\left(x_{i}-r\right)}^{2}}{n}}$$

where *x_i_* is the detected value, *r* is the expected value that corresponds to the reference axis, and *n* is the total number of detected values. The TSI has already been demonstrated to be a valid index of the dynamic postural control (Cè et al., 2018). In the DT conditions, the cognitive involvement in the two different postural tasks was assessed by considering the number of correct answers given by each participant. For both static and dynamic tests, DT cost was defined as the percentage change between ST and DT balance-related parameters. It was calculated as follows: [(DT − ST)/ST]*100.

### Statistical Analysis

For all the tests, the mean value among the three trials performed by each subject was considered for statistical analysis. The D’Agostino-Pearson test was employed to check the data normality distribution. An outlier analysis was performed to exclude subjects whose scores lie outside third quartile + 1.5*interquartile range and first quartile − 1.5*interquartile range. Pearson’s correlation was used to correlate static and dynamic postural balance parameters. Also, Pearson’s correlation was employed to analyze the correlation between handgrip strength and static/dynamic balance. The strength of the correlation was interpreted as follows: weak (*r* ≤ 0.35), moderate (0.36 < *r* < 0.67), high (0.68 < *r* < 0.90), and very high (*r* ≥ 0.90; Taylor, 1990). Since the output of the two devices was different, we employed two distinct paired *t*-tests to investigate differences between ST and DT conditions considering BS and BD, separately. A paired *t*-test was also used to compare the number of valid answers given in the BS-DT and in the BD-DT conditions. A one-way ANOVA for repeated measures was performed to compare the DT cost for each of the parameters considered. Values are expressed as mean ± standard error of the mean (SEM) and the significant level for differences was set at *p* < 0.05. Statistical analysis was performed using the software packages IBM SPSS Statistics for Windows (Version 24.0; IBMCorp., Armonk, NY). The effect sizes (Cohen’s *d*) following the paired *t*-test comparisons were calculated with G Power 3.1.5 (Faul et al., 2007). The magnitude of the effect size was interpreted as follows: 0.00–0.19: trivial; 0.20–0.59: small; 0.60–1.19: moderate; 1.20–1.99: large; and >2.00: very large (Hopkins et al., 2009). The choice of the sample size was based on an *a priori* power analysis. For the paired *t*-tests, we obtained a sample size of 44 subjects considering as input a large effect size (Cohen’s *d* = 0.5), *p* = 0.05, and a statistical power of 0.9. For the ANOVA, we obtained a sample size of 48 subjects, considering as input a large effect size (*f* = 0.25), *p* = 0.05, and a statistical power of 0.9. For Pearson’s correlation, we obtained a sample size of 37 subjects, considering as input a large effect size (Cohen’s *d* = 0.5), *p* = 0.05, and a statistical power of 0.9.

## Results

Fifty-seven out of the 61 enrolled subjects completed the trials and have been included in the data analysis for results; then, one subject was excluded after the outlier analysis. Table 1 reports the results of all parameters obtained from both static and dynamic postural balance tests.

**Table 1:** Results of the postural balance parameters in the single and dual-task condition.

	Single-task (ST)	Dual-task (DT)
Ellipse area (mm^2^)	135.00 ± 10.65	211.20 ± 23.07^§^
Sway path (mm/s)	11.70 ± 0.57	16.52 ± 1.07^§^
AP oscillations (mm)	21.23 ± 0.74	25.86 ± 1.23^§^
Total stability index	1.37 ± 0.16	3.02 ± 0.34^§^

§ Statistically significant (*p* < 0.001).

Data are presented as mean ± standard error of the mean (SEM).

The results of the correlational analysis are presented in Table 2. In detail, Pearson’s correlation revealed non-statistically significant correlations between TSI and all the static postural parameters (namely, ellipse area, sway path, and maximal AP oscillations). Moreover, Pearson’s correlation did not show any significant correlation between the static postural parameters and handgrip strength. On the contrary, a weak though significant negative correlation was found between TSI scores and handgrip strength test both in the ST (*p* < 0.05; *r* = −0.264) and in the DT (*p* < 0.05; *r* = −0.302) condition.

**Table 2:** Pearson’s correlations among parameters obtained from static postural balance test, dynamic postural balance test, and handgrip strength test.

**Single-task**	Ellipse area (mm^2^)	Sway path (mm/s)	AP oscillations (mm)	TSI
TSI	*r* = 0.169	*r* = 0.135	*r* = 0.202	
Handgrip strength (kg)	*r* = −0.006	*r* = 0.261	*r* = 0.120	*r* = −0.264^*^
**Dual-task**	Ellipse area (mm^2^)	Sway path (mm/s)	AP oscillations (mm)	TSI
TSI	*r* = −0.099	*r* = −0.008	*r* = −0.054	
Handgrip strength (kg)	*r* = −0.074	*r* = 0.198	*r* = 0.050	*r* = −0.302^*^

* Statistically significant (*p* < 0.05).

TSI, total stability index.

**Figure 2:**
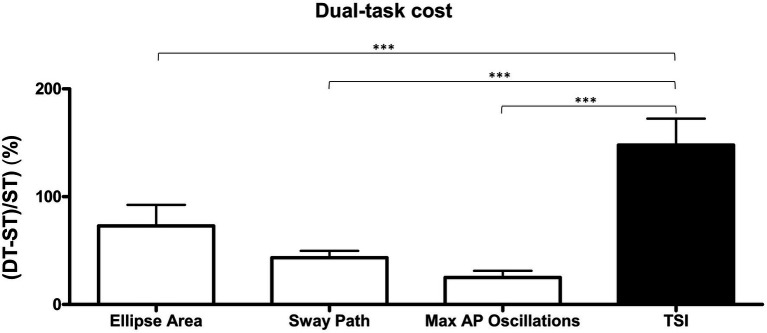
Dual-task cost for static and dynamic postural balance parameters. Black histogram represents the dynamic parameter (TSI, total stability index) while white histograms represent static parameters. DT, dual-task condition; ST, single-task condition. Data are presented as mean ± standard error of the mean (SEM). ***Significantly different (*p* < 0.001).

Paired *t*-tests showed an overall significant worsening of balance performance in the DT condition than in the ST condition, for all the parameters investigated (ellipse area: *p* < 0.001, Cohen’s *d* = 0.40; sway path: *p* < 0.001, Cohen’s *d* = 0.53; maximal AP oscillations: *p* < 0.001, Cohen’s *d* = 0.42; TSI: *p* < 0.001, Cohen’s *d* = 0.58) as shown in Table 1. The one-way ANOVA showed statistically significant differences in the DT cost (*p* < 0.001; *η_p_*^2^: 0.099) and the *post-hoc* multiple comparisons (Bonferroni test) showed a significantly higher (*p* < 0.001) DT cost for TSI (147.80 ± 24.55%) than ellipse area (72.93 ± 19.46%), sway path (43.45 ± 6.37%), and maximal AP oscillations (25.10 ± 24.55%), respectively (Figure 2). Additionally, the number of correct answers given by each participant was significantly lower (*p* < 0.001) in the BD-DT (4.03 ± 2.16) condition compared to the BS-DT (5.56 ± 0.42) condition.

## Discussion

The main purpose of this study was to compare the static and dynamic postural balance control in a group of older adults to understand whether these two conditions were interdependent from each other. Indeed, understanding whether a relationship between static and dynamic postural balance exists may lead to important practical applications in the assessment of postural balance control among older adults. Our results showed a non-significant correlation between static and dynamic postural balance control in any of the indexes investigated, both in ST and DT conditions. Although static and dynamic postural balance control is ruled by the same structures (i.e., cerebral cortex, basal ganglia, cerebellum, brainstem, and spinal cord), their different contribution in the two balance conditions (Takakusaki et al., 2017) could account for the non-significant correlations detected. Our findings are in line with previous researches where bipedal quiet stance showed no correlation with proactive (Timed Up & Go test and Functional Reach Test) and reactive (perturbed standing) balance (Muehlbauer et al., 2012).

Indeed, the human bipedal quiet stance has been modeled as a single inverted pendulum whose pivot is located at the ankle. In this model, the projection of the center of mass falls in front of the ankle, creating a dorsiflexor moment around the ankle, which is continuously counteracted by the stabilizing effect of tonic muscles (Morasso et al., 2019). This oscillation could be considered as a mostly automatic process of postural control since the subject is largely unaware of the adjustments of postural muscles (Takakusaki et al., 2017). Therefore, postural regulation mainly occurs at brainstem-spinal levels with neural circuits tuned by local loops of assistance or self-organized mechanisms due to the unperturbed and extremely predictable context (Lajoie et al., 1993). Conversely, when dynamic tasks are performed, continuous changes in the surrounding environment, acting forces, and sensory inputs happen, leading to a higher involvement of the cognitive process of postural control to achieve goal-directed movements (Takakusaki et al., 2017). Thus, a prevalence of the supra-spinal postural strategy is required due to the ongoing regulation of the movement for the adaptation to the new environment (Lajoie et al., 1993).

Our results on the addition of a cognitive-demanding task showed an overall decrease in postural balance performance under DT compared to ST condition, both in the static and dynamic assessment. This is not surprising since postural regulation could never be considered as totally automatic (Paillard, 2017a). Moreover, older adults exhibit less automatic processing of posture while standing, leading to greater involvement of cognitive resources (Boisgontier et al., 2013). Although the presence of a DT helped the subjects to address their attention to an external focus with a theoretical improvement of the balance performance (Masters and Maxwell, 2008), DT condition simultaneously resulted in increasing the complexity of the physiological and behavioral system. Consequently, an increase in information processing occurred, leading to cognitive-motor interference (Ghai et al., 2017).

The theoretical approaches for explaining this DT interference are 2-fold: the capacity sharing model and the bottleneck model (Pashler, 1994). In the first, it is assumed that people share a finite mental processing capacity among tasks. Thus, for each task that is performed, a section of this capacity is covered. When more than one task is performed, a decline of the performance on both tasks is registered if the total capacity is overcome. In the second model, it is postulated that when two tasks are performed, they compete for the same processing operation; consequently, a bottleneck occurs, and one or both tasks will be impaired.

**Figure 3:**
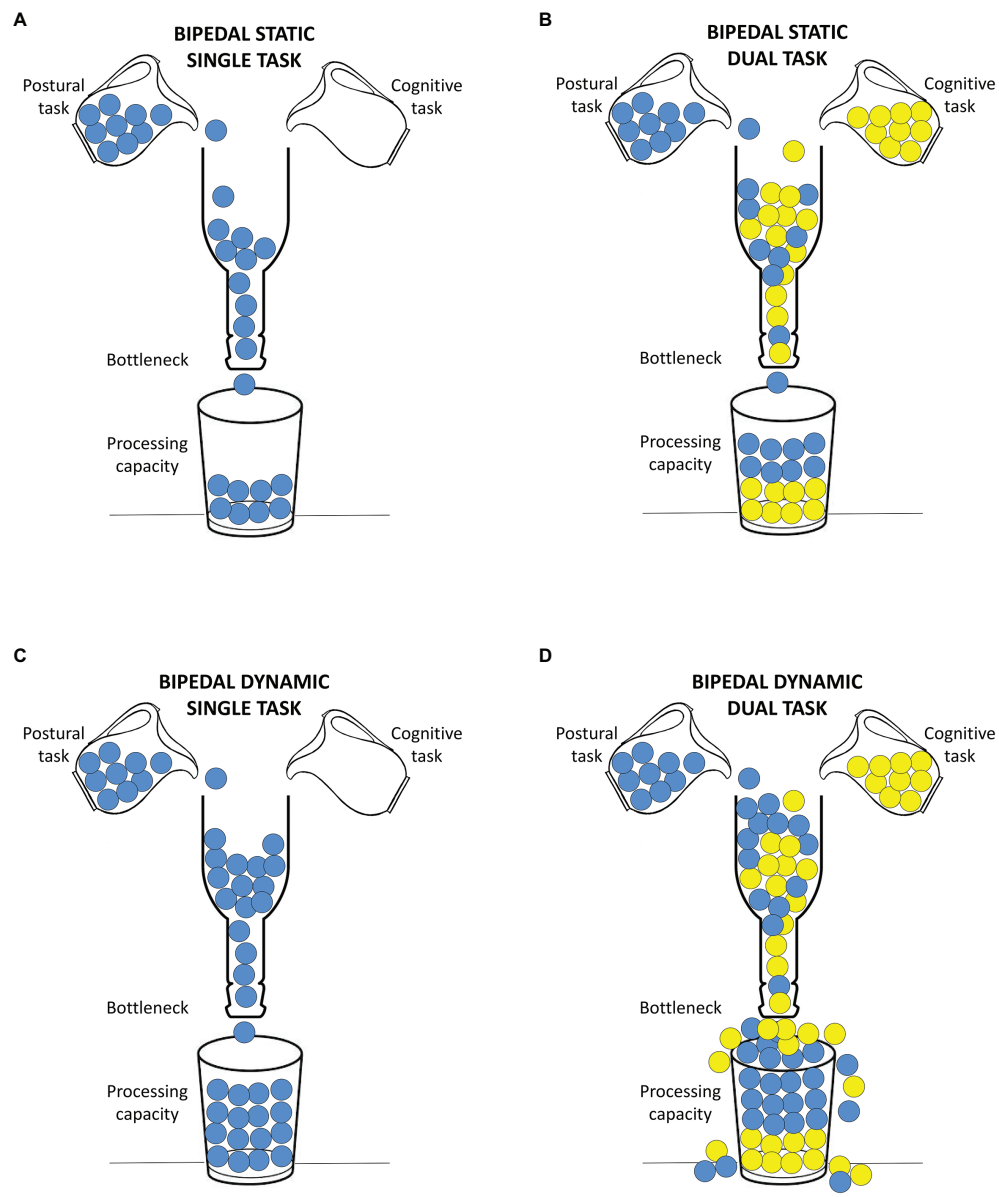
Capacity sharing and bottleneck model approaches to explain dual-task interference. **(A)** Inputs from the postural task (blue balls) cover a small part of the whole processing capacity (few balls in the glass) due to the mostly automatic control involved in quiet standing. No bottleneck occurs in this condition. **(B)** Under the static dual-task condition, a bottleneck results since postural (blue balls) and cognitive (yellow balls) tasks require the same mechanism at the same time. Moreover, the presence of both tasks covers a considerable part of the whole processing capacity (higher number of balls in the glass). **(C)** The dynamic postural task requires more processing capacity than static postural task (i.e., more blue balls in the glass). No bottleneck occurs in this condition. **(D)** Under the dynamic dual-task condition, a bottleneck occurs as for **(B)** and the processing capacity required, exceeds its total amount (the balls overflow the glass).

Since the two theoretical approaches are not mutually exclusive (Pashler, 1994), they could together account for the greater worsening of postural balance (+147.8%) and cognitive (+38%) performance (i.e., less correct answers given) detected under dynamic than static condition (Figure 3). During the dynamic test (Figure 3C), the voluntary control of postural balance required a greater processing capacity than in the static test (Figure 3A; Woollacott and Shumway-Cook, 2002). Being the cognitive task of the same difficulty in both the balance conditions (Figures 3B,D), the older adults should have invested a higher mental processing capacity in the dynamic postural task without worsening the cognitive task. However, the performance of the cognitive task decreased as well: supposedly, the processing capacity required to cope with the dynamic test in the DT condition exceeded the overall available capacity. Finally, since the cognitive and the postural task required the same central mechanism simultaneously, the resulting bottleneck contributed, together with the processing capacity saturation, to the highest worsening of both tasks in the dynamic condition (Figure 3D). Conversely, the bottleneck model could explain alone the worsening of the static postural performance (+25.10%, +43.45%, and +72.93% for ellipse area, sway path, and maximal AP oscillations, respectively). Indeed, the static postural task was less demanding (i.e., less capacity required; Figure 3A) and allowed to cover each task without exceeding the total amount of the processing capacity (Figure 3B).

A difference between static and dynamic postural balance control has been detected looking at the handgrip test results. An intriguing significant weak correlation was found between handgrip strength and dynamic postural balance control. This correlation seems to demonstrate that the greater the strength of the older adults, the better the dynamic postural balance performance (i.e., lower TSI). Although this relationship certainly deserves further investigation, our data are in line with the results published by Forte et al. (2014), who pointed out the interaction between strength and dynamic postural balance (Forte et al., 2014). However, although the level of strength has often been related to fall-risk, its relationship with balance performance is still a debated issue in the scientific literature since some authors found no relationship between postural sway and strength or power (Ringsberg et al., 1999; Muehlbauer et al., 2012).

It is relevant to mention some limitations of the present work. Postural balance control is a multifactorial construct in older adults. Although we enrolled a homogeneous group of healthy non-faller older adults thanks to the physiatric screening, we could not control all the variables that might have affected balance performance, and thus, few of them could have been neglected, confounding some of our findings. Then, although we followed the recommendations of the international society of posturography for the static test standardization, the employment of the same feet position could have been challenging depending on the anthropometric characteristics of each subject. Last, the interpretation of the DT interference on brain structures and thus on balance performance followed a theoretical approach. Further studies measuring brain involvement while performing different balance tasks are warranty to support the interpretation presented in this study.

In conclusion, although the same structures govern static and dynamic postural balance control, the relative contribution of each structure is different in the two balance conditions. The absence of significant correlations supports this consideration, corroborating the assumption that postural balance assessments should include both static and dynamic conditions in older adults. Moreover, concurrently cognitive-interference tasks exacerbated the degradation of postural control performance, especially under dynamic conditions. Therefore, static and dynamic postural control assessments with cognitive tasks are encouraged to give clinicians more accurate indications on postural control for the development of tailored training programs.

## Data Availability Statement

The raw data supporting the conclusions of this article will be made available by the authors, without undue reservation.

## Ethics Statement

The study involving human participants was reviewed and approved by the Human Ethical Committee of the Department of Biomedical Sciences of the University of Padova (n° HEC-DSB/08-18). The participants provided their written informed consent to participate in this study.

## Author Contributions

GM, AR, and AP conceived and designed the experiments, and wrote the paper. AR, GM, MA, and FV performed the experiments and analyzed the data. AP contributed the materials. All authors contributed to the article and approved the submitted version.

### Conflict of Interest

The authors declare that the research was conducted in the absence of any commercial or financial relationships that could be construed as a potential conflict of interest.
